# Neuroprotective Role of Atractylenolide-I in an In Vitro and In Vivo Model of Parkinson’s Disease

**DOI:** 10.3390/nu9050451

**Published:** 2017-05-02

**Authors:** Sandeep More, Dong-Kug Choi

**Affiliations:** Department of Biotechnology, College of Biomedical and Health Science, Konkuk University, Chungju 27478, Korea; sandeepbcp@gmail.com

**Keywords:** Atractylenolide-I, astrocyte, microglia, neuroinflammation, Parkinson’s disease

## Abstract

Parkinson’s disease (PD) is an age-related neurological disorder characterized by a loss of dopaminergic neurons within the midbrain. Neuroinflammation has been nominated as one of the key pathogenic features of PD. Recently, the inadequate pharmacotherapy and adverse effects of conventional drugs have spurred the development of unconventional medications in the treatment of PD. The purpose of this study is to investigate the anti-neuroinflammatory mechanisms of Atractylenolide-I (ATR-I) in in vivo and in vitro models of PD. Nitrite assay was measured via Griess reaction in lipopolysaccharide (LPS) stimulated BV-2 cells. mRNA and protein levels were determined by a reverse transcription-polymerase chain reaction (RT-PCR) and immunoblot analysis, respectively. Further, flow cytometry, immunocytochemistry, and immunohistochemistry were employed in BV-2 cells and MPTP-intoxicated C57BL6/J mice. Pre-treatment with ATR-I attenuated the inflammatory response in BV-2 cells by abating the nuclear translocation of nuclear factor-κB (NF-κB) and by inducing heme oxygenase-1 (HO-1). The intraperitoneal administration of ATR-I reversed MPTP-induced behavioral deficits, decreased microglial activation, and conferred protection to dopaminergic neurons in the mouse model of PD. Our experimental reports establish the involvement of multiple benevolent molecular events by ATR-I in MPTP-induced toxicity, which may aid in the development of ATR-I as a new therapeutic agent for the treatment of PD.

## 1. Introduction

Parkinson’s disease (PD) is a progressive, neurodegenerative disorder characterized by the loss of dopaminergic neurons in the substantia nigra pars compacta (SNpc) and microglial malfunction. Accumulating evidence from various scientific studies suggests that neuroinflammation is one of the important factors involved in the selective degeneration of the nigral neurons in PD [1,2,3,4]. Extracts from medicinal plants and their secondary metabolites have conventionally been used to treat numerous clinical diseases, including inflammation-associated diseases [5]. Atractylenolide-I (ATR-I) is one of the major bioactive ingredients that have been isolated from the rhizomes of *Atractylodes macrocephala* (*A. macrocephala*). *A. macrocephala* is also popularly known as “*Baizhu*” in traditional Chinese medicine and has long been used for a variety of corresponding medicinal properties [6]. This species of plant belongs to the Asteraceae family, and its distribution has been found in some Asian countries, such as Japan, China, and Korea. ATR-I, chemically a sesquiterpene, is a highly lipophilic volatile lactone. In terms of the activities elicited by ATR-I in a biological system, pharmacological studies have demonstrated its gastrointestinal inhibitory effects [7], anti-oxidant activity [8], and anti-cancer activity [9]. ATR-I has been reported to increase the hunger and mid-arm muscle circumference of patients with cachectic cancer, and it can significantly inhibit serum levels of interleukin-1 (IL-1), tumor necrosis factor-α (TNF-α), and urine proteolysis-inducing factors [10]. Several reports have illustrated that ATR-I can selectively antagonize the toll-like receptor-4 or the membrane-bound glucocorticoid receptor, to elicit an anti-inflammatory effect [11,12]. Apart from the bioactivities reported for ATR-I, a systematic and detailed examination of its anti-inflammatory mechanism has not yet been performed. Additionally, the chronic use of existing symptomatic treatment is often associated with debilitating side effects [13] and none seem to halt the progression of PD. So far, the development of effective anti-neuroinflammatory therapies has been impeded by our limited knowledge of the pathogenesis of PD.

Based on these existing evidences, the purpose of our present study is to unravel the mechanisms responsible for the anti-neuroinflammatory effect of ATR-I in LPS- and MPTP-induced neuroinflammatory PD models in BV-2 cells and C57BL/6J mice, respectively. Our results demonstrate the involvement of multiple molecular events mediated by ATR-I for its anti-neuroinflammatory effect in LPS-induced BV-2 cells and MPTP-induced neurotoxicity. Our results may aid in the development of ATR-I as a new therapeutic agent for controlling inflammation associated with PD.

## 2. Materials and Methods

### 2.1. Material

ATR-I (purity ≥ 98%) was purchased from BaoJi Herbest Bio-Tech Co., Ltd. (Baoji, China). LPS (*Escherichia coli* 0111:B4, Sigma, St. Louis, MO, USA), MPP^+^, MPTP, Tween-20, bovine serum albumin, dimethyl sulfoxide (DMSO), sodium nitrite, sulfanilamide, *N*-(1-naphthyl) ethylenediamine dihydrochloride, and zinc protoporphyrin IX (ZnPP-IX) were purchased from Sigma. 2,7-dichlorodihydrofluorescein diacetate (H_2_DCFDA) was obtained from Invitrogen. The 6-well and 96-well tissue culture plates and 100 mm culture dishes were purchased from Nunc Inc. (Aurora, IL, USA). Fetal bovine serum (FBS) was purchased from PAA Laboratories Inc. (Etobicoke, ON, Canada). Dulbecco’s modified Eagle medium (DMEM) and phosphate buffer saline (PBS), as well as other cell culture reagents, were obtained from Gibco/Invitrogen (Carlsbad, CA, USA). The protease-inhibitor cocktail tablets and phosphatase-inhibitor cocktail tablets were supplied by Roche (Indianapolis, IN, USA). Antibodies for NF-κB/p65 and nucleolin were obtained from Santa Cruz Biotechnology (Santa Cruz, CA, USA), and the antibodies for inducible nitric oxide synthase (iNOS), ERK, p-ERK, Akt, p-Akt, PI3K, p-PI3K, the inhibitor of kappa B-alpha (IκB-α), p-IκB-α, and β-actin were supplied by Cell Signaling Technology (Danvers, MA, USA). Antibodies for heme oxygenase (HO-1) and manganese superoxide dismutase (MnSOD) were procured from Enzo Life Sciences (Farmingdale, NY, USA). The following vendors were used for the procurement of other antibodies: macrophage-1 antigen (Mac-1) (AbD Serotec), glial fibrillary acidic protein (GFAP) (Abcam, Cambridge, UK), and tyrosine hydroxylase (TH) (Calbiochem, MA, USA). All of the other chemicals used in this study are of analytical grade and were obtained, unless otherwise noted, from Sigma Chemical Co. (St. Louis, MO, USA).

### 2.2. BV-2 Cell Culture

The BV-2 immortalized murine microglial cell line [14] was provided by Dr. Kyungho Suk, Kyungbook National University. The method used for culturing the BV-2 cells was followed according to our previously published report [15]. ATR-I was dissolved in DMSO. The control/vehicle group was treated with only DMSO.

### 2.3. Animals and MPTP Administration

**Scheme 1:**
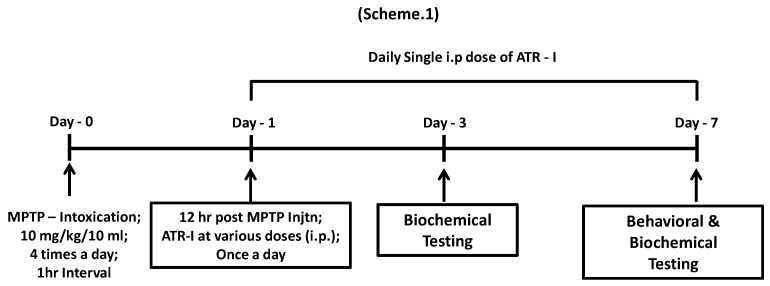
Experimental design illustrating the treatment and testing schedule for animal experiments.

Male C57BL6/J mice (age, 8–9 weeks; weight, 25–28 g) (Samtako Bio Korea, Gyeonggi-do, Korea) were acclimatized for 14 days, prior to the drug treatment. Animal experiments and the experimental procedures were approved by the Institutional Animal Care and Use Committee of Konkuk University. The animals were housed in a controlled environment (23 °C ± 1 °C and 50% ± 5% humidity) and were allowed food and water *ad libitum*. The room’s lights were switched on between 8:00 a.m. and 8:00 p.m. One hundred and twenty six animals were randomly divided into the following seven groups, comprised of 18 animals each: Vehicle group, MPTP group, ATR-I (30 mg/kg/10 mL) group, Selegiline (10 mg/kg/10 mL) + MPTP, ATR-I (3 mg/kg/10 mL) + MPTP group, ATR-I (10 mg/kg/10 mL) + MPTP group, and ATR-I (30 mg/kg/10 mL) + MPTP group. As demonstrated in the experimental design (Scheme 1), a 10 mg/kg/10 mL MPTP was administered intraperitoneally (i.p.) on four occasions, at an interval of 1 h [16,17], to all of the animals except for those in the vehicle group and ATR-I (30 mg/kg/10 mL) i.e., per se group. Twelve hours after the last MPTP injection, ATR-I (i.p.) or selegiline (i.p.) was administered once daily, for three and seven days. The animals were sacrificed on day three and day seven. The MPTP was freshly prepared in saline before use. ATR-I was suspended in 0.5% methyl cellulose solution containing 1% Tween 80, before dosing. The control/vehicle group was administered as plain methyl cellulose solution.

### 2.4. Pole Test

To measure the degree of bradykinesia, a typical symptom of Parkinsonism, a pole test was performed with a slightly modified reported procedure [18]. This test was successively performed three times for each mouse. The experimenter was kept uninformed to the experimental groups.

### 2.5. Nitrite Measurement Assay

The release of nitric oxide (NO) was measured using the Griess reaction in the cell supernatant [19]. BV-2 cells (2.5 × 10^4^ cells/mL) were seeded in 96-well plates in 200 µL culture medium, pre-treated with three different doses of ATR-I (25 μM, 50 μM, and 100 μM) for 1 h, and were then stimulated with or without LPS (100 ng/mL) for 24 h. The further procedure was carried out in accordance with our previously published data [15]. The results are representative of three independent experiments.

### 2.6. Immunohistochemistry

The procedure was followed according to our previously published data [20], with slight modifications. The free-floating brain sections from striatum (STR) and SNpc from the mice sacrificed on day three and seven post-MPTP injection were tagged with primary antibodies, including anti-Mac-1 (1:500; AbD Serotec, Oxford, UK) and an anti-TH antibody (1:1500; Calbiochem San Diego, CA, USA), respectively. The samples were visualized by a DAB Peroxidase (HRP) Substrate Kit (Vector Laboratories, CA, USA) following the manufacturers protocol. Fluorescence staining was performed with the primary antibodies of anti-GFAP (1:2000; Abcam), incubated overnight at 4 °C. The sections were then incubated with rabbit antigoat GFAP secondary antibody (1:200) (Alexa Flour 488, Invitrogen, Carlsbad, CA, USA) for 1 h. Stained cells were viewed using a bright field and fluorescence microscope (Carl Zeiss). The quantification of the effects in the brain-tissue sections was performed by using Image J software (Bethesda, MD, USA) to count the number of TH-positive cells in SNpc and STR, respectively. Data were presented as a percentage of the control-group values.

### 2.7. Isolation of Total RNA and Reverse Transcription-Polymerase Chain Reaction (RT-PCR)

ATR-I at varying concentrations was pre-incubated with BV-2 cells (5.0 × 105 cells/mL) for 1 h, and was then treated with or without LPS (100 ng/mL) for 6 h. The animal tissues were washed in cold 0.1 M PBS (pH 7.4) and homogenized using a tuberculin syringe with TRIzol, and were subsequently stored at −80 °C until RNA extraction. The total RNA was isolated by extraction with TRIzol (Invitrogen). For RT-PCR, 2.5 μg of the total RNA from each group was reverse transcribed using a First-Strand cDNA Synthesis kit (Invitrogen). The cDNA was then amplified by PCR using specific primers as mentioned previously [15]. The following primers were used for PCR, iNOS: F-5′-CTTGCAAGTCCAAGTCTTGC-3′ and R-5′-GTATGTGTCTGCAGATGTGCTG-3′; TNF-α: F-5′-TTCGAGTGACAAGCCTGTAGC-3′ and R-5′-AGATTGACCTCAGCGCTGAGT-3′; IL-6: F-5′-GGAGGCTTAATTACACATGTT-3′ and R-5'-TGATTTCAAAGATGAATTGGAT-3‘; IL-1β: F-5′-CATATGAGCTGAAAGCTCTCCA-3′ and R-5′-GACACAGATTCCATGGTGAAGTC-3′; MCP-1: F-5′-AGATGCAGTTAACGCCCCAC-3′ and R-5′-GACCCATTCCTTCTTGGGGT-3′; MMP-9: F-5′-CGCTCATGTACCCGCTGTAT-3′ and R-5′-TGTCTGCCGGACTCAAAGAC-3′; HO-1: F-5′-GGATTTGGGGCTGCTGGTTTC-3′ and R-5′-GCAGTGGCAAAGTGGAGATTG-3′; Mac-1: F-5′-TCAAGCGGCAGTACAAGGAC-3′ and R-5′-GCCACACACAGAGCTTGCTT -3′; GFAP: F-5′-TTCCTGTACAGACTTCTCC-3′ and R-5′-CCCTTCAGGACTGCCTTAGT-3′; GAPDH: F-5′-GCAGTGGCAAAGTGGAGATTG-3′ and R-5′-TGCAGGATGCATTGCTGACA-3′. The PCR products were analyzed on 1% agarose gels stained with ethidium bromide, and the bands were visualized by UV light.

### 2.8. SDS-PAGE and Western Blot Analysis

BV-2 cells (5.0 × 10^5^ cells/mL) were cultured in 6-well plates and were pre-treated for 1 h with respective doses of ATR-I, followed by exposure to LPS (100 ng/mL) for 30 min or 18 h, respectively. The preparation of cell lysates (whole and nuclear), electrophoresis, and the immunoblotting procedures were followed as mentioned previously [15]. For the animal experiment, STR and ventral midbrain (VM) tissues were processed according to our previously published data [21]. PVDF membranes were incubated overnight with anti-iNOS (1:1000), anti-IκB-α (1:1000), anti-p-IκB-α (1:1000), anti-ERK (1:1000), anti-p-ERK (1:1000), anti-Akt (1:1000), anti-p-Akt (1:1000), anti-PI3K (1:1000), anti-p-PI3K (1:1000), anti-HO-1 (1:1000), anti-β-actin (1:2000), anti-MnSOD (1:1000), anti-GFAP (1:50,000), anti-Mac-1 (1:500), anti-TH (1:1000), anti-NF-κB/p65 (1:500), and anti-nucleolin (1:500), followed by a 1 h incubation with horseradish peroxidase-conjugated secondary antibodies (1:1000–5000) (Cell Signaling Technology and Santa Cruz biotechnology). The optical densities of the antibody-specific bands were analyzed with a Luminescent Image Analyzer, LAS-3000 (Fuji, Tokyo, Japan).

### 2.9. Detection of Intracellular ROS Generation Using a Flow Cytometer

Intracellular reactive oxygen species (ROS) production was measured using the non-fluorescent compound H_2_DCFDA, as previously described [22], with slight modifications. H_2_DCFDA is a stable nonpolar compound that readily diffuses into cells to produce DCFH. Intracellular ROS in the presence of peroxidase changes DCFH to the highly fluorescent compound DCF; therefore, the amount of ROS is proportional to the fluorescent intensity exhibited by the cells [23]. Briefly, cells (5.0 × 10^5^ cells/well in 6-well plate) were incubated with vehicle (DMSO) or ATR-I (25 µM, 50 µM, and 100 µM) at 37 °C for 1 h, before being stimulated with LPS for another 4 h. Furthermore, cells were treated with 10 μM of H_2_DCFDA (dissolved in DMSO) for 45 min at 37 °C. The cells were trypsinized and the fluorescent intensity was analyzed with a FACS Calibur flow-cytometry system.

### 2.10. Immunofluorescence Assay

BV-2 cells (5 × 10^5^ cells/well) were cultured on sterile cover slips in a 24-well plate and treated with ATR-I (100 µM) for 1 h, followed by LPS (100 ng/mL) for 0.5 h, to detect the intracellular p65 sub-unit of NF-κB. Further procedure for the immunofluorescence assay was followed in accordance with the previously reported method [15].

### 2.11. Statistical Analyses

Statistical analyses were performed using GraphPad software version 5 (GraphPad, La Jolla, CA, USA). Data are the mean ± standard error of mean (SEM) of at least three independent experiments. Significant differences between the groups were determined using a one-way analysis of variance (ANOVA) followed by Tukey’s post-hoc analysis. The *p* values < 0.05 are considered statistically significant.

## 3. Results

### 3.1. ATR-I Suppresses LPS-Induced NO Release in BV-2 Cells

**Figure 1:**
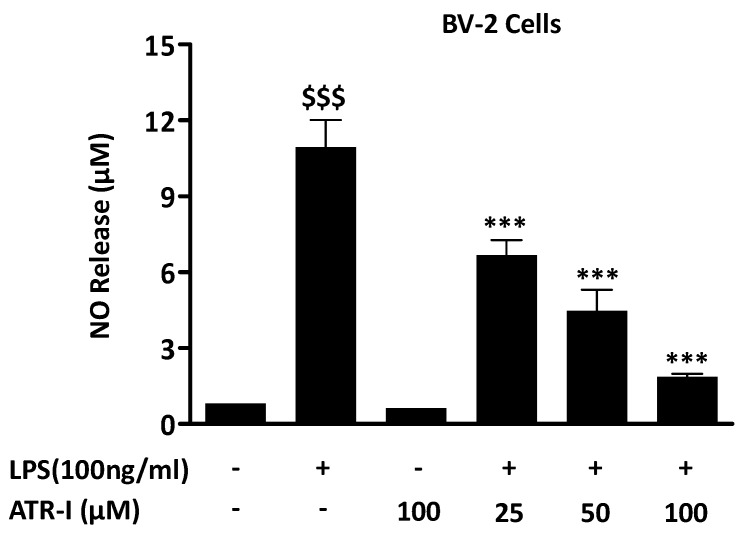
NO release was measured in BV-2 cells (*n* = 6) as mentioned in “Material and Methods” section. ^$$$^ *p* < 0.001 vs. vehicle group and *** *p* < 0.001 vs. LPS group.

LPS exposure to BV-2 cells from the control group and only ATR-I group did not exhibit any increase in NO levels. Pre-treatment with ATR-I at 25 μM, 50 μM, and 100 μM significantly reduced the NO levels in LPS-stimulated BV-2 cells, in a dose-dependent manner (Figure 1A).

### 3.2. ATR-I Diminishes iNOS, TNF-α, IL-6, IL-1β, MCP-1, and MMP-9 mRNA-Expression in LPS-Stimulated BV-2 Cells

**Figure 2:**
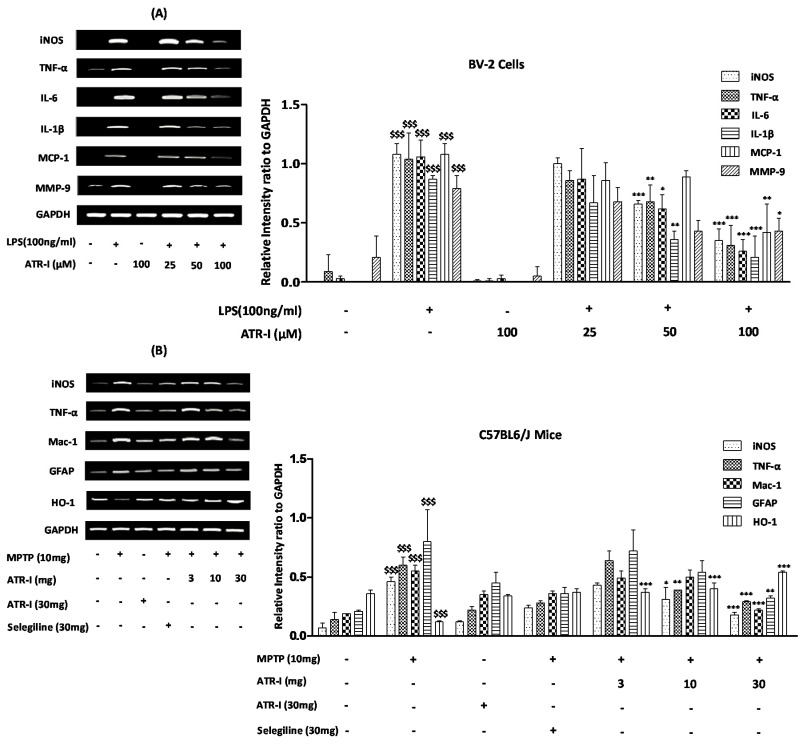
Quantification data are shown in the right panel. (**A**) mRNA levels (*n* = 6) of iNOS, TNF-α, IL-6, IL-1β, MCP-1, MMP-9 in BV-2 cells and (**B**) mRNA levels (*n* = 4) of iNOS, TNF-α, Mac-1, GFAP, and HO-1 in mice. GAPDH was used as an internal control. ^$$$^ *p* < 0.001 vs. vehicle group; * *p* < 0.05, ** *p* < 0.01, and *** *p* < 0.001 vs. LPS and MPTP-treated group.

The mRNA levels of TNF-α, IL-6, IL-1β, monocyte chemotactic protein-1 (MCP-1), and matrix metallopeptidase-9 (MMP-9) were increased 6 h after LPS treatment (Figure 2A). Further, ATR-I was observed to suppress the LPS-induced mRNA expression of iNOS, TNF-α, IL-6, and IL-1β, in a dose-dependent fashion. However, the inhibitory effects of ATR-I on the mRNA expression of MCP-1 and MMP-9 were not observed to be dose-dependent.

### 3.3. ATR-I Diminishes mRNA-Expression of Microglia-Derived Pro-Inflammatory Mediators and Induces HO-1 Expression in MPTP-Induced Model of Neuroinflammation

MPTP significantly upregulated the mRNA expression of iNOS, TNF-α, GFAP, and Mac-1, and decreased the expression of HO-1 in mice STR. Post-treatment with ATR-I significantly inhibited the mRNA expressions of iNOS, Mac-1, GFAP, and TNF-α in the MPTP-intoxicated mice (Figure 2B). This inhibitory effect of ATR-I was dose-dependent for the mRNA expressions of iNOS, TNF-α, and GFAP. Furthermore, post-treatment with ATR-I significantly and dose-dependently increased the mRNA expression of HO-1 in MPTP-intoxicated mice (Figure 2B).

### 3.4. ATR-I Diminishes the Microglial and Astroglial Markers in Striatum of MPTP-Intoxicated Mice

**Figure 3:**
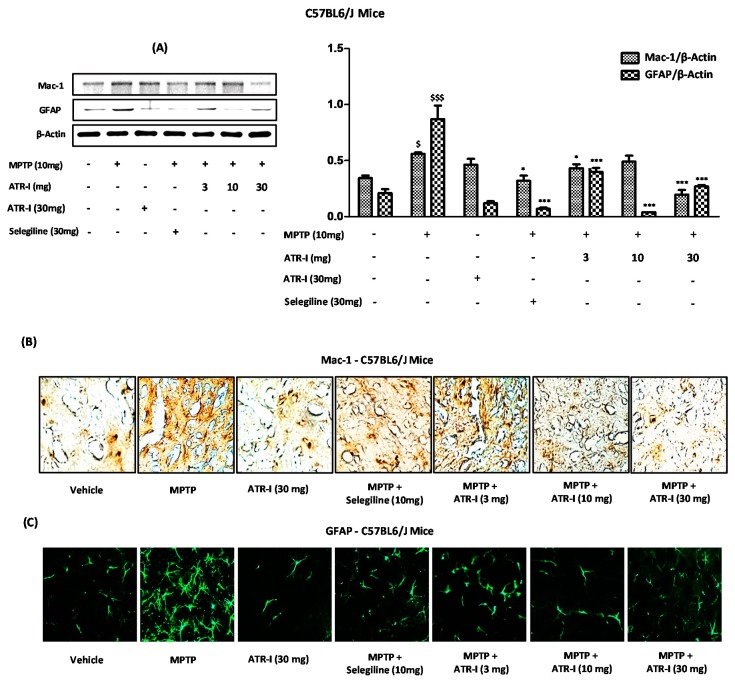
(**A**) Western blot for Mac-1 and GFAP expression in the STR (*n* = 3). ^$^ *p* < 0.05 and ^$$$^ *p* < 0.001 vs. vehicle group; * *p* < 0.05, and *** *p* < 0.001 vs. MPTP-treated group; (**B**) Representative image presenting Mac-1-positive microglia in the STR of MPTP-intoxicated mice (*n* = 4); (**C**) Representative image presenting GFAP-positive astroglia in the STR of MPTP-intoxicated mice (*n* = 4).

Figure 3A depicts the protein content of Mac-1 and GFAP, which was significantly increased in the STR after MPTP exposure, compared to the vehicle group. However, post-treatment with ATR-I significantly decreased the elevated protein levels of Mac-1 and GFAP. On the other hand, ATR-I did not elicit a dose-dependent inhibition of GFAP, as observed in protein blots (Figure 3A). Similarly, results from the staining of the STR revealed that microglial cells from the STR of the vehicle group did not exhibit significant staining. Both the Mac-1 (Figure 3B) (brown) and GFAP (Figure 3B) (green) staining signal significantly increased on day three. Post-treatment with ATR-I mitigated the significantly increased number of Mac-1 and GFAP microglia and astrocytes. Furthermore, these results also synchronized with the immunoblot analysis (Figure 3A), wherein ATR-I effectively abated the Mac-1 and GFAP expressions in the STR of the MPTP-intoxicated mice.

### 3.5. ATR-I Subsides LPS- and MPTP-Induced Increases of iNOS Expression and Modulates LPS-Induced Phosphorylation of ERK, PI3K, and Akt

**Figure 4:**
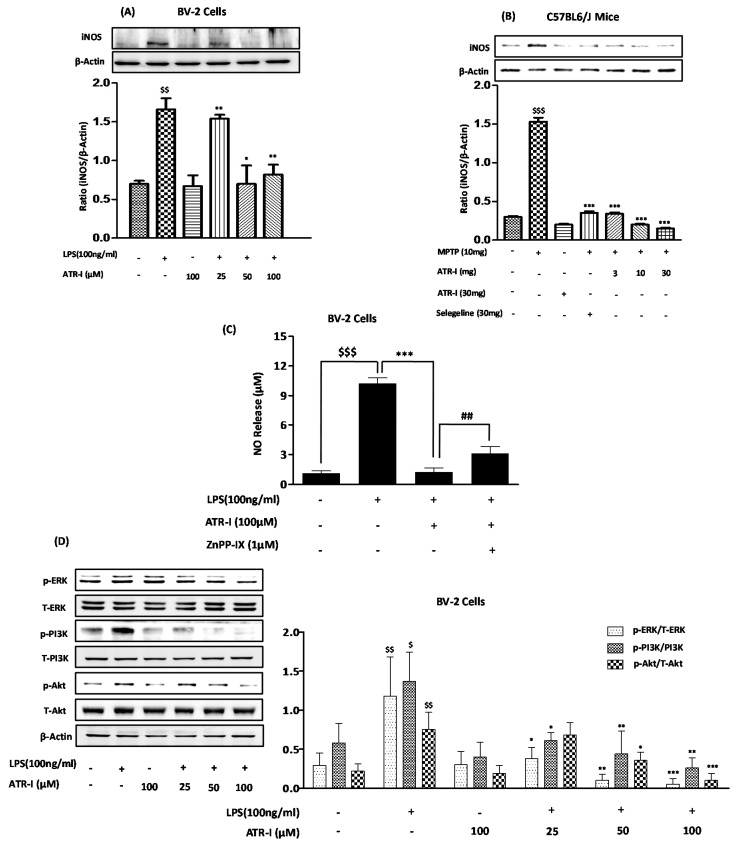
Quantitative protein analysis (*n* = 6) of iNOS (**A**,**B**) and (**D**) p-ERK-MAPK, p-PI3K, and p-Akt in BV-2 cells. ^$$$^ *p* < 0.001, ^$$^ *p* < 0.01, and ^$^ *p* < 0.05 vs. vehicle group; * *p* < 0.05, ** *p* < 0.01, and *** *p* < 0.001 vs. LPS and MPTP-treated groups. (**C**) NO release was measured in BV-2 cells (*n* = 6) as mentioned in “Material and Methods”. ^$$$^ *p* < 0.001 vs. vehicle group; *** *p* < 0.001 vs. LPS-treated group; *^##^ p* < 0.01 vs. ATR-I + LPS treated group.

Exposure to LPS (Figure 4A) and MPTP (Figure 4B) significantly increased the protein expression of iNOS in BV-2 cells and MPTP-intoxicated mice, respectively. However, treatment with ATR-I at different doses reduced the protein expression of iNOS. ATR-I strongly and dose-dependently inhibited ERK phosphorylation (Figure 4D), whereas ATR-I had no effect on p38 and c-Jun N-terminal kinase (JNK). LPS exposure significantly increased PI3K and Akt phosphorylation in contrast to the control. Furthermore, pretreatment with ATR-I dose-dependently and significantly inhibited the phosphorylation levels of PI3K and Akt, respectively (Figure 4D). However, ATR-I treatment alone did not alter the phosphorylation levels of PI3K and Akt in BV-2 cells.

### 3.6. HO-1 Mediates the Inhibitory Effect of ATR-I on NO Release in LPS-Stimulated BV-2 Cells

ATR-I (100 µM), in combination with LPS, was able to decrease NO release in BV-2 cells (Figure 4C). At 1 μM, ZnPP-IX reversed the inhibition of LPS-stimulated NO production caused by pre-treatment with ATR-I. These data indicate that HO-1 mediates the anti-inflammatory effects of ATR-I in BV-2 cells (Figure 4C).

### 3.7. ATR-I Ameliorates the Degradation of IκB-α and Inhibits Nuclear Translocation of NF-κB in LPS and MPTP Model of Neuroinflammation

**Figure 5:**
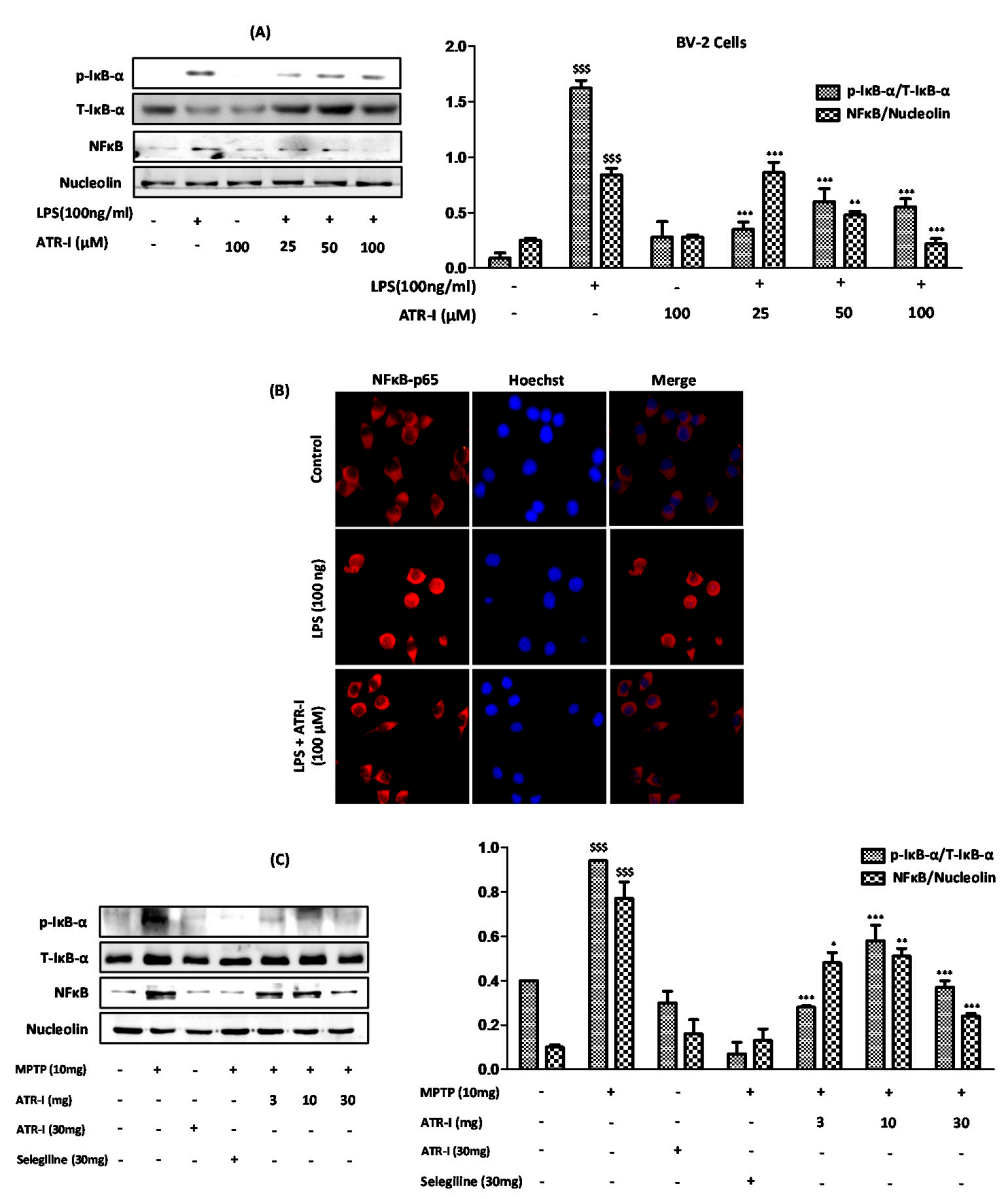
(**A**) Nuclear and whole-cell extracts (*n* = 3) were prepared and analyzed by Western blot to asses (**A**) anti-p-IκB-α and NF-κB/p65; (**B**) The sub-cellular location of the NFκB-p65 subunit was determined by an immunofluorescence assay (*n* = 3). The total nuclear and whole-cell lysates (*n* = 4) of the STR were tagged with (**C**) anti-p-IκB-α and NF-κB/p65. ^$$$^ *p* < data compared with the vehicle group; * *p*, ** *p*, *** *p* < data compared with LPS and MPTP-treated groups.

Exposure to LPS (Figure 5A) and MPTP (Figure 5C) significantly increased the protein expressions of p-IκB-α; however, ATR-I significantly reversed the effects of LPS and MPTP on IκB-α phosphorylation, but this effect was not dose-dependent in either of the models. Furthermore, ATR-I significantly inhibited the LPS- and MPTP-induced nuclear translocation of NF-κB/p65, as determined by Western blotting and the immunofluorescence assay (Figure 5B).

### 3.8. ATR-I Suppresses the LPS-Induced Production of ROS in BV-2 Cells, While Upregulates the Anti-Oxidant Defense System in the MPTP-Induced Model of Neuroinflammation

**Figure 6:**
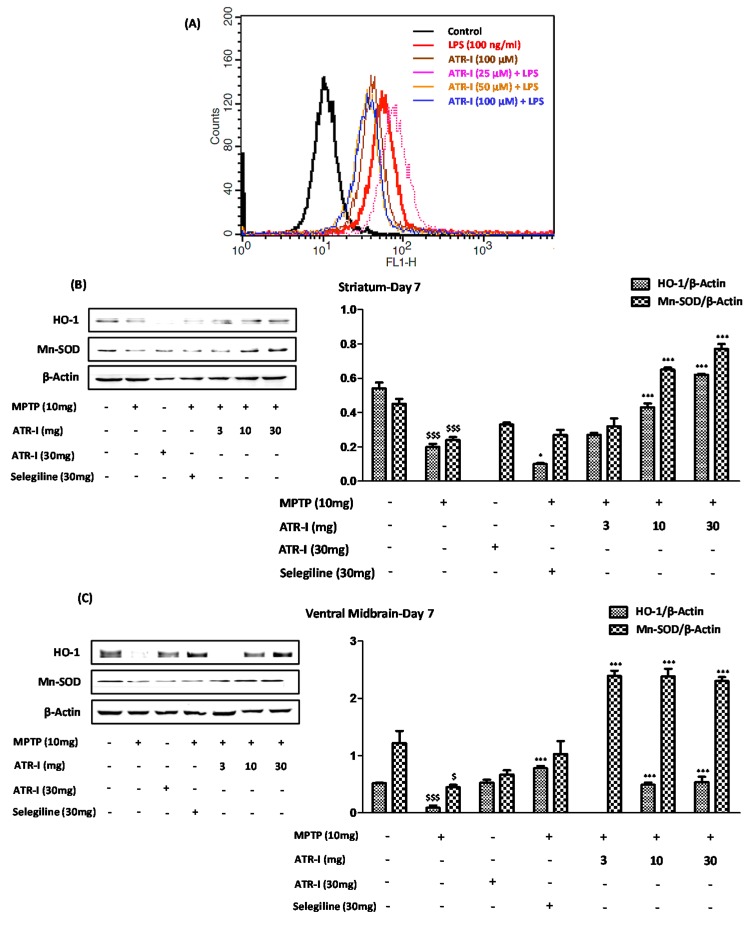
(**A**) The histogram obtained by fluorometric analysis portrays one representative set of results (*n* = 6) from the three different experiments in BV-2 cells; (**B**) Represents protein levels for HO-1 and MnSOD in STR and (**C**) VM. A densitometric analysis of HO-1 and MnSOD is shown in the right panel. ^$^ *p* < 0.05 and ^$$$^ *p* < 0.001 vs. vehicle group; * *p* < 0.05 and *** *p* < 0.001 vs. MPTP-treated group.

As indicated in Figure 6A, the BV-2 cells that were exposed to LPS exhibited a prominent curve shift toward the right, indicating significant ROS generation. Furthermore, the incubation of the BV-2 cells with ATR-I at varying doses significantly reduced the ROS content, as seen by the curve shift towards the left. The lower dose of 25 µM did not result in a significant reduction of ROS content, and instead exhibited a greater extent of ROS release compared to the LPS exposure, which might have been due to a sample variation. The remaining two doses (50 μM and 100 μM), however, had a similar effect, wherein the LPS-induced ROS release was suppressed in the BV-2 cells. In line with these reports, our results from animal experiments indicate that MPTP significantly decreased the protein levels of HO-1 and MnSOD in the STR (Figure 6B) and VMs (Figure 6C) of the mice, as compared to the vehicle group. However, ATR-I was able to reverse the decreased protein levels of HO-I in the STR and VM, in a dose-dependent fashion.

### 3.9. ATR-I Mitigates MPTP-Induced Dopaminergic Neurodegeneration and Motor Dysfunction in a Mouse Model of Neuroinflammation

**Figure 7:**
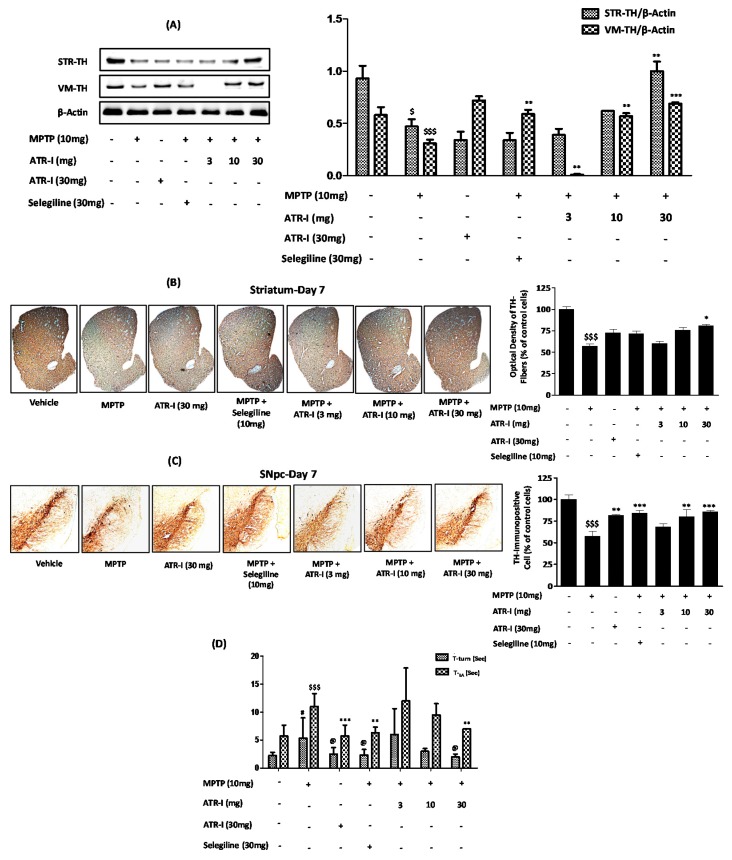
Protective effects of ATR-I against MPTP in STR and VM. Western blot for TH protein expression in STR and VM (**A**); Representative coronal images of TH-positive striatal fibers (**B**,**C**) SNpc neurons. Densitometric analysis for TH protein expression is provided in the right panel (*n* = 4). ^$$$^ *p* < 0.001 and ^$^ *p* < 0.05 vs. vehicle group; * *p* < 0.05, ** *p* < 0.01, and *** *p* < 0.001 vs. MPTP-treated group; (**D**) The Pole test was carried out on day 7 (*n* = 4). ^$$$^ *p* < 0.001 and ^#^ *p* < 0.05 vs. vehicle group; ^@^ *p* < 0.05, ** *p* < 0.01, and *** *p* < 0.001 vs. MPTP-treated group.

MPTP intoxication significantly decreased the levels of TH expression in the STR and VM (Figure 7A). The MPTP + ATR-I (10 mg/kg, and 30 mg/kg) treatment groups presented a greater extent of TH expression than the MPTP-treated group, in both the STR and VM tissues. However, the TH expression of STR and VM tissues in the 3 mg/kg ATR-I treated mice was lower than those in the MPTP-treated mice. Only treatment with ATR-I did not have an effect on TH expression in the STR, whereas the TH expression in the VM was significantly increased. On the other hand, selegiline was not effective in the reversal of TH expression in the STR; however, selegiline significantly increased TH expression in the VM.

Similarly, as displayed in the representative photographs (Figure 7), numerous TH-positive fibers in STR- and TH-positive neurons in SNpc were observed in the vehicle group. Exposure to MPTP decreased the TH-positive fiber (56.97% ± 4.23%) in the STR (Figure 7B) and the TH-positive cells (57.75% ± 6.89%) in the SNpc, to a significant extent (Figure 7C). ATR-I alone did not modify the TH-positive neurons, as shown by the stain intensities of the STR and SNpc. ATR-I prevented the MPTP-induced loss of TH-positive fibers in the STR (Figure 7B) (60.18% ± 4.50%, 75.78% ± 4.98%, and 80.86% ± 2.56%) and SNpc neurons (Figure 7C) (68.31% ± 6.11%, 80.24% ± 14.22%, and 86.14% ± 1.76%), in a dose-dependent manner.

MPTP intoxication developed bradykinesia, as was confirmed by the turning on the top and the climbing down the pole; however, post-treatment with ATR-I and selegiline significantly decreased the T-turn and T-LA time taken by the mice (Figure 7D).

## 4. Discussion

The role of sesquiterpene lactones in the inhibition of the central transcription factor NF-κB is well documented [24]. Sesquiterpene lactones indicate the active principle of many of the anti-inflammatory drugs used in traditional medicine. Furthermore, observations have shown that they also possess other bioactivities for hematomas, contusions, sprains, and rheumatic diseases [25]. ATR-I has been documented for a variety of pharmacological uses in eastern Asian countries regarding the treatment of edema, anorexia, splenic asthenia, excessive perspiration, and abnormal fetal movement [26,27]. Moreover, previous findings also advocate the in vivo and in vitro anti-inflammatory activities of ATR-I’s [12,28,29,30].

We used LPS to induce inflammatory pathology in BV-2 cells as they simulate the inflammatory response that occurs in neurodegenerative diseases [31] and MPTP to induce neuroinflammation in C57BL6/J mice, because they replicate a variety of important pathobiochemical features of PD [32,33]. Growing evidence from a variety of studies suggests that proinflammatory factors released from activated microglia play an important role in the initiation and progress of neurodegenerative processes, including PD [34,35]. NO, produced from iNOS, is a proinflammatory factor and nitrosative stress source. [36]. Microglia in PD have been observed to densely grow in the striatum and SNpc with increased expression of proinflammatory mediators, including TNF-α, IL-1β, and IL-6 [37]. Furthermore, during inflammatory conditions, chemokine MCP-1 acts to cause the migration of specific leukocyte sets to inflammation sites, while MMP-9 plays a vital role by degrading the blood-brain barrier [38,39]. Hence, we first investigated the ability of ATR-I to curb the release of NO and proinflammatory mediators. We showed that ATR-I significantly decreased the mRNA expression profile of inflammatory mediators, MCP-1 and MMP-9, in our models. Our data corresponds with a previous report, wherein ATR-I also inhibited NO and TNF-α [40]. Gabriel and coworkers have established that iNOS plays an essential role in the neurotoxic process initiated by MPTP and suggested that inhibitors of iNOS may provide a protective benefit in the treatment of PD [41]. Recent experimental data has designated the dopaminergic system in the midbrain as the most susceptible area following MPTP-mediated inflammatory damage that is caused by an over-activation of astrocytes and microglia [42,43].

Microglia are resident macrophage cells in the brain, that promptly react in response to toxins in their microenvironment and quickly proliferate, become hypertrophic, and persistently increase the expression of a large number of marker molecules, such as Mac-1 and TNF-α [44], and are further transformed to macrophage-like cells in patients with PD [45]. GFAP is a chief intermediate astrocyte filament protein that mediates the interaction of astrocytes with other cells that are essential for the formation and maintenance of myelin. Therefore, GFAP has received much attention as a reliable biomarker in studies of central nervous system diseases [46]. Astrocytes anticipate brain injury via a process termed “reactive gliosis” [47], and the density of GFAP-positive astrocyte is negatively correlated with the reduction of dopaminergic neuron [48]. Therefore, molecules that can modulate microglial and astrocyte activation may potentially exert neuroprotective effects against MPTP. Hence, apart from iNOS, we also chose to study Mac-1 in microglia and GFAP in astroglia, as they are specific markers that represent micrcoglia and astroglia activation [49]. Our experimental data in both in vivo and in vitro studies demonstrated that ATR-I treatment inhibits LPS/MPTP-induced iNOS expression. Also, data from our immunohistochemistry and Western blot studies indicate that the systemic administration of ATR-I diminished the MPTP-induced production of GFAP and Mac-1 in the microglial cells of the STR; however, we did not observe any significant effect of ATR-I on the GFAP and Mac-1 profiles in the microglial cells of the SNpc. Our results are in coherence with earlier reports, wherein the mitigation of microglial inflammation provides neuroprotection in an MPTP model of PD [20].

Recently, it was reported that the activation of the PI3K/Akt pathway is involved in the LPS-induced expression of iNOS [27,50]. Also, an accumulating body of evidence indicates the PI3K/Akt pathway’s role in the inflammatory response of LPS, whereby NF-κB is activated through IκB degradation [51,52]. We found that ATR-I significantly and dose-dependently blocked the phosphorylation of PI3K and Akt in response to an LPS challenge. LPS is involved in the stimulation of a variety of intracellular signaling pathways in the microglia that involve MAPKs and NF-κB [53,54]. Furthermore, MAPKs facilitate the gene expression of many inflammatory mediators [55,56].

We found that ERK and Akt are specific targets of ATR-I. Earlier reports suggested the importance of ERK-signaling in the production of pro-inflammatory cytokines, while NF-κB mediates the gene expression of proinflammatory cytokines and additional effector molecules [57]. Pharmacological interference in NF-κB’s transcriptional activity may therefore represent an important therapeutic strategy for the mitigation of neuroinflammatory diseases that are linked to oxidative stress. Our data demonstrate that ATR-I inhibits the phosphorylation of the IκB-α protein, thereby disrupting the activation of NF-κB and its nuclear translocation, in response to LPS/MPTP-induced neuroinflammation. A number of recent reports demonstrate that the mitochondrial malfunctioning and increase in ROS leads to neuronal-cell demise via apoptosis [58,59]. Additionally, historical data has also indicated the role of ROS in the activation of NF-κB [60,61]. In the present study, pre-treatment with ATR-I significantly attenuated intracellular ROS generation in BV-2 cells that were exposed to LPS. HO-1 is an anti-inflammatory cytoprotective protein which protects against oxidative stress [62,63]. Recently, several experiments have suggested that HO-1 induction is involved in the inhibition of LPS-induced NO production [64,65]. Our experiments involving ZnPP-IX (specific HO-1 inhibitor) revealed that the induction of HO-1 caused by pre-treatment with ATR-I in LPS-stimulated BV-2 cells, is responsible for abating LPS-induced NO release. Apart from HO-1, MnSOD found exclusively in the mitochondrial matrix [66], plays a vital role in neutralizing ROS [67]. The over-expression of MnSOD is reported to protect cells and attenuate MPTP-induced toxicity [68]. Our present data indicates that the anti-inflammatory effects of ATR-I are, at least in part, due to the inhibition of ROS and the induction of antioxidant enzymes (HO-1 and MnSOD). Our results are also in agreement with previous works, wherein the induction of HO-1 facilitates cellular protection against NO-induced injury [69,70] and MPTP-induced oxidative stress [71,72,73].

As a rate-limiting enzyme, TH is required for catecholamine synthesis and is a hallmark indicator for evaluating the neuroprotective ability of compounds in PD, since a decrease of TH activity is positively correlated with PD severity [74]. We found ATR-I to restore the population of surviving dopaminergic neurons in the STR and SNpc, in response to MPTP intoxication, thus asserting the neuroprotective effect of ATR-I in the MPTP model of PD. The pole test data revealed the protective benefits of ATR-I for mitigating bradykinesia against MPTP. Our data is in coherence with earlier published data, wherein the maintenance of normal motor behavior is positively correlated with the preservation of TH-positive neurons [20]. Additionally, data concerning the toxicity and efficacy profile of ATR-I in different models of PD would produce more reliable data. This data would further help in sustaining the applicability of ATR-I in clinical studies and also its use in PD.

## 5. Conclusions

In conclusion, the present study showed significant anti-neuroinflammatory efficacy of ATR-I in the MPTP-induced model of neuroinflammation, through increasing HO-1 and MnSOD levels, along with an increase in the counts of TH-immunoreactive neurons. Additionally, ATR-I exhibits promising efficacy in the LPS-induced model of PD. Taken together, the results indicate that ATR-I may be an effective anti-neuroinflammatory agent to be considered as a lead candidate against PD.
